# Fabrication of Poly(acrylic acid)/Boron Nitride Composite Hydrogels with Excellent Mechanical Properties and Rapid Self-Healing Through Hierarchically Physical Interactions

**DOI:** 10.1186/s11671-018-2800-2

**Published:** 2018-12-05

**Authors:** Shishan Xue, Yuanpeng Wu, Meiling Guo, Dan Liu, Tao Zhang, Weiwei Lei

**Affiliations:** 1 School of Materials Science and Engineering, Southwest Petroleum University, Chengdu, 610500 China; 2 State Key Laboratory of Oil and Gas Reservoir Geology and Exploitation, Southwest Petroleum University, Chengdu, 610500 China; 3 Institute for Frontier Materials, Deakin University, Locked Bag 20000, Geelong, Victoria 3220 Australia; 4 College of Textile and Clothing Engineering, Soochow University, Suzhou, 215123 China

**Keywords:** Self-healing hydrogel, Boron nitride nanosheets, Hierarchically physical interactions

## Abstract

Many living tissues possess excellent mechanical properties and self-healing ability. To mimic these living tissues, a series of novel composite hydrogels, poly(acrylic acid)/surface-modified boron nitride nanosheets (PAA/BNNS-NH_2_) were fabricated simply through hierarchically physical interactions: molecular-scale metal coordination interaction between –COOH of PAA and Fe^3+^ and nanoscale H-bond between –COOH of PAA and –NH_2_ of BNNS-NH_2_. The composite hydrogels exhibit both excellent mechanical properties (including enhanced fracture stress, elongation, toughness, Young’s modulus, and dissipated energy) and rapid healing ability without any external stimulus. Especially, the B_0.5_P_70_ (the hydrogel with BNNS concentration of 0.5 mg mL^− 1^, the water content of 70 wt%) exhibits a fracture stress of ~ 1311 kPa and toughness of ~ 4.7 MJ m^− 3^, almost ~ 3 times and ~ 8 times to B_0_P_70_, respectively. The excellent properties, combined with the simple preparation method, endow these composite hydrogels with potential applications.

**Electronic supplementary material:**

The online version of this article (10.1186/s11671-018-2800-2) contains supplementary material, which is available to authorized users.

## Background

Hydrogels with three-dimensional networks formed by covalent bonds and/or physical interactions crosslinking containing a large amount of water possess high hydrophilicity, water-holding capacity and unexceptional biocompatibility [1–4], enabling to be one of the most popular biomaterials. However, most hydrogels have poor mechanical property, which largely limited the applications. It is well known that many living tissues, such as muscle, ligament, and skin, possess excellent mechanical property and significant ability to heal wounds autonomously [5–7]. Inspired by these living tissues, materials with high mechanical properties and self-healing ability have been explored for various applications [8, 9], including tissue engineering, drug release, wound dressing, contact lenses, sensors, and actuators [2, 10–12]. Ihsan et al. reported a polyampholyte hydrogel self-healed through re-forming the ironic bonds at fracture surface [7]. Zhang et al. designed a PVA self-healable hydrogel with fast self-healing process through hydrogen bonds [13]. Tao et al. prepared a cold resistance self-healing hydrogel crosslinked by dynamic catechol-borate ester bonding which enable to self-heal at both room temperature and low temperature [14]. However, all these self-healable materials have a common weakness: poor mechanical property [15–19] largely limited the applications.

In order to improve the mechanical property of the hydrogels, some inorganic nanomaterials have been introduced to the crosslinked systems. Han et al. reported a supermolecular hydrogel by using graphene oxide nanosheets to reduce the temperature for self-healing [15]. Si et al. exploited a new ultrahigh-water-content, super-elastic, and shape-memory nanofiber-assembled hydrogels [20]. The flexible SiO_2_ nanofibers were introduced to enhance mechanical property and to accelerate shape memory and pressure response. Especially, Duan et al. developed poly(vinyl alcohol)/boron nitride nanosheet (PVA/BNNS) composite hydrogels with enhanced mechanical properties [21]. Gao et al. fabricated a nanocomposite hydrogel filled with exfoliated montmorillonite which dramatically improved the fracture elongation [22]. Zhong et al. designed graphene oxide (GO)/poly(acrylic acid) (PAA/GO) nanocomposite hydrogels which significantly enhanced the mechanical properties [23]. Novel composite self-healing hydrogels with enhanced mechanical property are still highly pursued although exploited hydrogels have advanced significantly in recent years. Boron nitride nanosheets, “white graphene”, exhibit many excellent properties including superb mechanical properties, extraordinary chemical inertness, and remarkable non-toxicity [24–26]. Notably, surface-modified BN nanosheets served as nanofillers in the nanocomposite hydrogels enhance mechanical and thermal properties and have been reported in recent works [27, 28]. Therefore, the development of a novel composite hydrogel with surface-modified BN nanosheets is still highly pursued.

Here, the novel composite hydrogels are fabricated from poly(acrylic acid) (PAA) and amino groups surface-modified boron nitride nanosheet (BNNS-NH_2_) through hierarchically physical interactions: molecular-scale metal coordination interaction between –COOH of PAA and ferric ion (Fe^3+^) and nanoscale H-bond between –COOH and BNNS-NH_2_ were reported. The introduction of BNNS-NH_2_ enhanced the mechanical property and accelerated self-healing process of the hydrogels. This work provides a new route to prepare hydrogels with excellent mechanical properties and rapid self-healing ability.

## Method/Experimental

### Materials

Potassium persulfate (KPS; 99.0%) and FeCl_3_**·**6H_2_O (99.0%) were purchased from J&K Chemical Technology, and acrylic acid (AA; 98.0%) was purchased from Sigma-Aldrich. All these chemicals were used as received without any purification. Rhodamine B (95.0%) was purchased from Sigma-Aldrich. BNNS-NH_2_ was obtained by our previous work [24]. Deionized water was used throughout the experiments.

### Preparation of BNNS-NH_2_ Dispersion

BNNS-NH_2_ was prepared according to our previous work [24]. In order to make BNNS-NH_2_ steadily dispersed in the polymer network, it is indispensable to prepare the BNNS-NH_2_ water dispersions. To obtain the stable BNNS-NH_2_ dispersions, magnetic stirring and ultrasound bath were utilized at room temperature. The BNNS-NH_2_ dispersions with concentration of 1.0, 0.8, 0.5, and 0.1 mg mL^− 1^ were obtained by the following procedure. The 100 mg, 80 mg, 50 mg, and 10 mg of BNNS-NH_2_ were added in 100 mL of deionized water, respectively, under magnetic stirring (1000 rpm) for 24 h at room temperature in air ambient to obtain mixtures, and then the mixtures were sonicated (20 kHz) at room temperature for 2 h in air ambient to get stable dispersions. For prohibiting loss of the water solution, the obtained dispersions were preserved in sealed vessels with different marks for following preparation of self-healing hydrogels.

### Preparation of Self-Healing Hydrogel

PAA as the common polymer with abundant –COOH groups enables to establish the amount of intrachain and interchain hydrogen bonds which endow the polymer to possess superior elasticity and favorable strength [29]. In addition, metal coordination interactions are set up between –COOH of PAA and ferric ion (Fe^3+^). The two kinds of reversible non-covalent bonds equipped the hydrogel with self-healing property. The hydrogels crosslinked by non-covalent bonds always possess inferior mechanical properties. In order to enhance the strength of the hydrogel, BNNS-NH_2_ was introduced to the polymer three-dimensional network, which established hydrogen bonds between –NH_2_ of BNNS-NH_2_ and –COOH of PAA. Here, the composite PAA/BNNS-NH_2_ hydrogels were abbreviated as B_x_P_y_, in which B represents BNNS-NH_2_, x is the content of the BNNS-NH_2_ (mg mL^− 1^), P means PAA/BNNS-NH_2_ composite hydrogel, and y refers to the water content of the PAA/BNNS-NH_2_ composite hydrogel (mass fraction, wt%). The hydrogels were prepared according to a procedure described below. Typically, 10 mL of AA, 0.25 g of FeCl_3_**·**6H_2_O (1.05 mol% of AA), and 0.1 g of KPS (0.25 mol% of AA) were dissolved in BNNS-NH_2_ dispersions with different concentrations or deionized water under magnetic stirring (1000 rpm) at room temperature for 10 min under air ambient to form a homogeneous mixture. After that, N_2_ was bubbled into the mixture to remove oxygen (10 min), and then polymerization was carried out at 25 °C in water bath for 6 h to form hydrogels. Hydrogels prepared as aforementioned procedure and parameters from BNNS-NH_2_ dispersions with the concentration of 1.0, 0.8, 0.5, and 0.1 mg mL^− 1^ were denoted as B_1_P_90_, B_0.8_P_90_, B_0.5_P_90_, and B_0.1_P_90_, respectively, while hydrogels prepared from deionized water was named as B_0_P_90_.

It is well known that the hydrogels with different water contents possess entirely different mechanical properties. In order to characterize the influence of water content to the mechanical properties of the hydrogels, the hydrogels with different water contents were prepared as follows. Firstly, the B_x_P_90_ hydrogels were prepared as the aforementioned procedure and parameters. Then, the as-prepared B_x_P_90_ hydrogels were exposed in air at room temperature for different times depending on the final water content of the hydrogels. Thereinto, the obtained drying hydrogels with different water contents were labeled as B_x_P_70_, B_x_P_50_, and B_x_P_25_, respectively. The water content was calculated by the formula: water content = *W*_w_/*W*_t_, where the *W*_w_ is the weight of the water and *W*_t_ is the whole weight of the hydrogel. On the other hand, the crosslinking densities of B_x_P_90_ hydrogels were calculated from the results of rheological measurements, and it is well known that the higher crosslinking density leads to the more robust mechanical property. To verify the theory, it is indispensable to carry out the tensile tests. However, the B_x_P_90_ hydrogels were so soft that the electrical universal material testing machine cannot recognize the sample exhibiting no load, so the composite hydrogels with lower water content were highly required to fabricate. The hydrogels with different water contents were cut into different shapes or sizes for the following various tests.

### Mechanical Test

In order to characterize the mechanical properties of the hydrogels, the as-prepared hydrogels were cut into a flaky shape (50 mm × 2 mm × 2 mm) and tested by the electrical universal material testing machine with a 200 N load cell under a speed of 50 mm min^− 1^ at 25 °C and a humidity of approximately 45%. The tensile stress (*σ*) representing strength was calculated by the equation: *σ* = *F*/(*a* × *b*), where *F*, *a*, and *b* were force of loading and width and thickness of hydrogels, respectively. The strain (*ε*) representing stretchability was defined as the change of the length, illustrated by the formula: *ε* = (*l* − *l*_0_)/*l*_0_ × 100%, where *l* and *l*_0_ represent the lengths after and before testing, respectively. Stiffness was characterized by Young’s modulus which was obtained from the slope of the stress-strain curve at the low strains. The toughness of the samples was illustrated as the area under stress-strain curves. The cyclic tensile tests were performed at the same experimental condition which aimed to obtain the dissipated energy. The dissipated energy was characterized by the area between the loading-unloading curves and *X*-axis.

### Characterization

The Fourier-transform infrared (FTIR) spectra was carried out to record the samples’ FTIR characters, which were recorded on a Thermo Scientific Nicolet 6700 spectrometer in attenuated total reflection (ATR) mode, with a resolution of 4 cm^− 1^ within the range 400–4000 cm^− 1^. The morphology of the hydrogels after the frozen drying process was observed on scanning electronic micrographs (SEM, Carl Zeiss AG, ZEISS EV0 MA15). In order to analyze the viscoelasticity of the hydrogels and calculate the crosslinking density, the rheological measurements were carried out by using a rheometer (HAAKE MARS III Thermo Fisher Scientific Limited, China) to measure the storage moduli (G’) and loss moduli (G”). The tensile tests were carried out to analyze the mechanical properties of the samples, which were conducted using an electrical universal material testing machine with a 200 N load cell (Instron 2360).

## Results and Discussion

**Scheme. 1:**
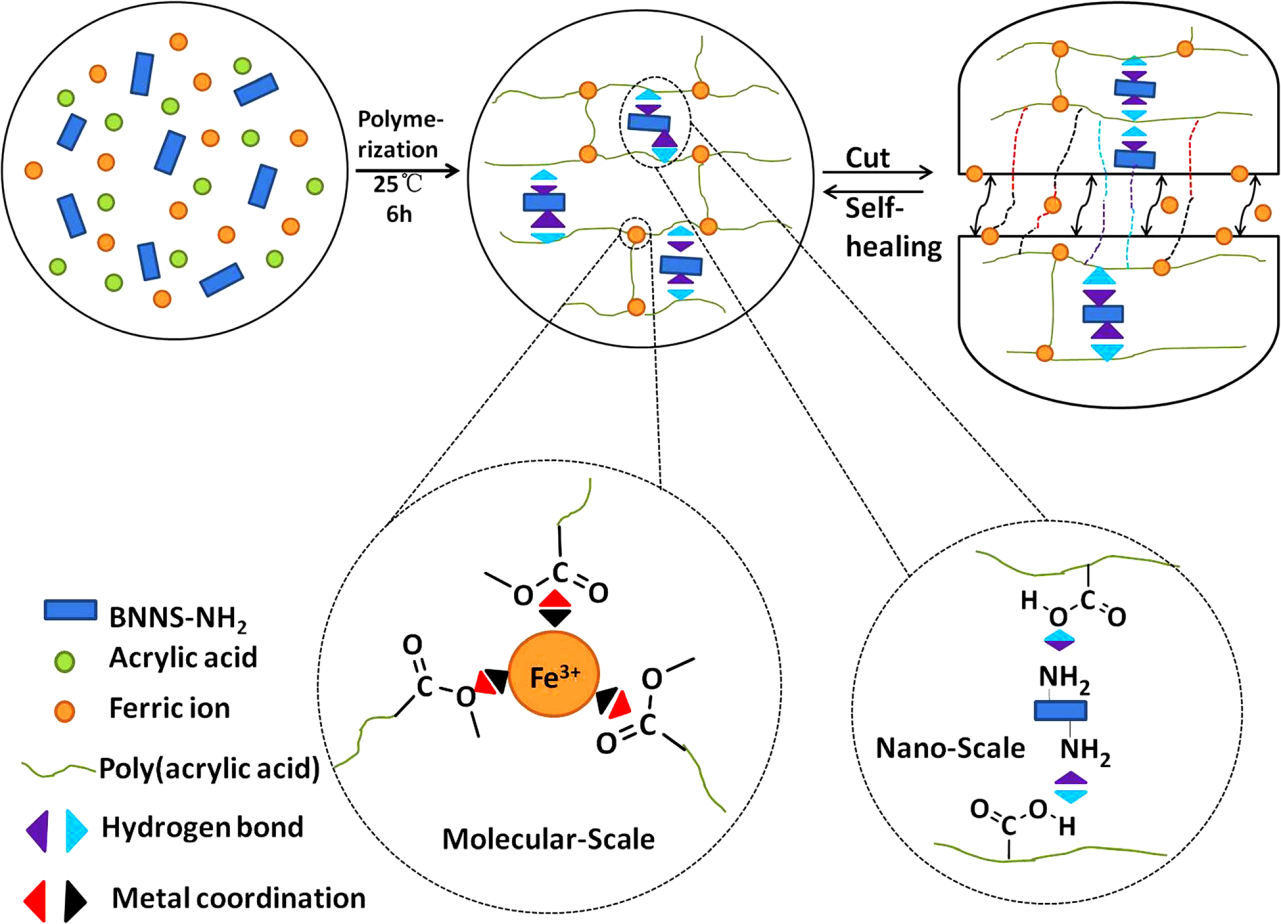
Scheme illustrating the formation of a PAA/BNNS-NH_2_ composite hydrogel with self-healing ability

The PAA/BNNS-NH_2_ composite hydrogels were simply formed by in situ polymerization of AA, with the presence of Fe^3+^ and the BNNS-NH_2_. The as-formed PAA macromolecular chains were crosslinked by hierarchically physical interactions: metal coordination interaction between carboxyls (–COOH) of the PAA and Fe^3+^ in molecular scale, and hydrogen bond interaction between –COOH of the PAA and –NH_2_ of BNNS-NH_2_ in nanoscale, resulting in the formation of three-dimensional networks (Scheme 1).

**Fig. 1:**
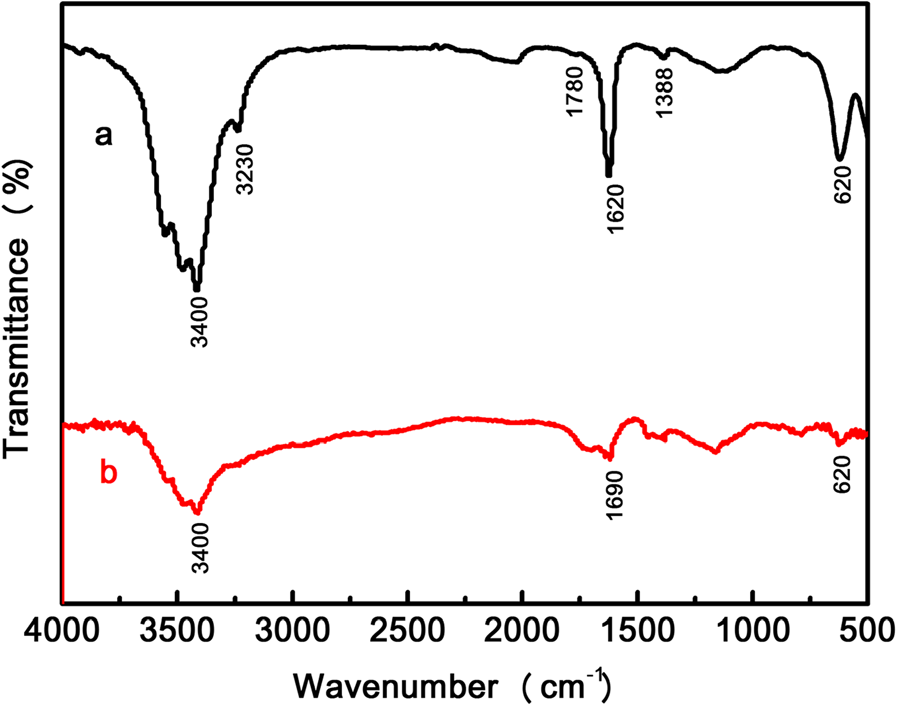
FTIR spectra of **a** a composite hydrogel and **b** a PAA hydrogel

The two different types of physical interactions within the PAA/BNNS-NH_2_ composite hydrogels were revealed by FTIR spectroscopy firstly. From the results in Fig. 1, PAA shows a characteristic stretching (–C=O stretching) at 1690 cm^− 1^, which has shifted to 1620 cm^− 1^ in the PAA/BNNS-NH_2_ composite hydrogel. This shift indicates the existence of hydrogen bond interactions between –COOH of PAA and –NH_2_ of BNNS-NH_2_ [30, 31]. The presence of hydrogen bond can be verified by the fact that the characteristic peaks at 3400 cm^− 1^for –COOH became less obvious in the composite hydrogel [32, 33]. The peak at 3230 cm^− 1^ can be assigned to N–H stretching vibration in the composite hydrogel. Metal coordination interaction was revealed by the peak at 620 cm^− 1^ in both PAA hydrogel and composite hydrogel, demonstrating that metal coordination interaction between Fe^3+^ and –COO^−^ was formed in the network system [34]. The in-plane B–N stretching at 1388 cm^− 1^ and the out-of-plane B-N-B bending vibrations at 1780 cm^−1^can be seen from composite hydrogel (Fig. 1a), confirming the presence of BNNS-NH_2_.

**Fig. 2:**
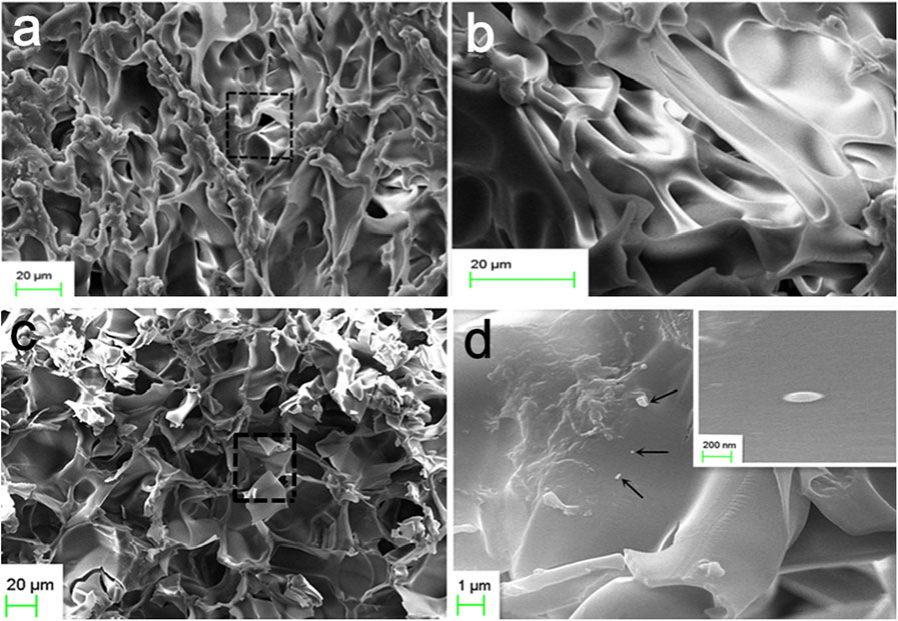
SEM images of **a**, **b** PAA hydrogel and **c**, **d** composite hydrogel. The BNNS-NH_2_ was pointed out by arrows in (**d**), and the inserted image in (**d**) is the zoomed-in image of the BNNS-NH_2_

**Fig. 3:**
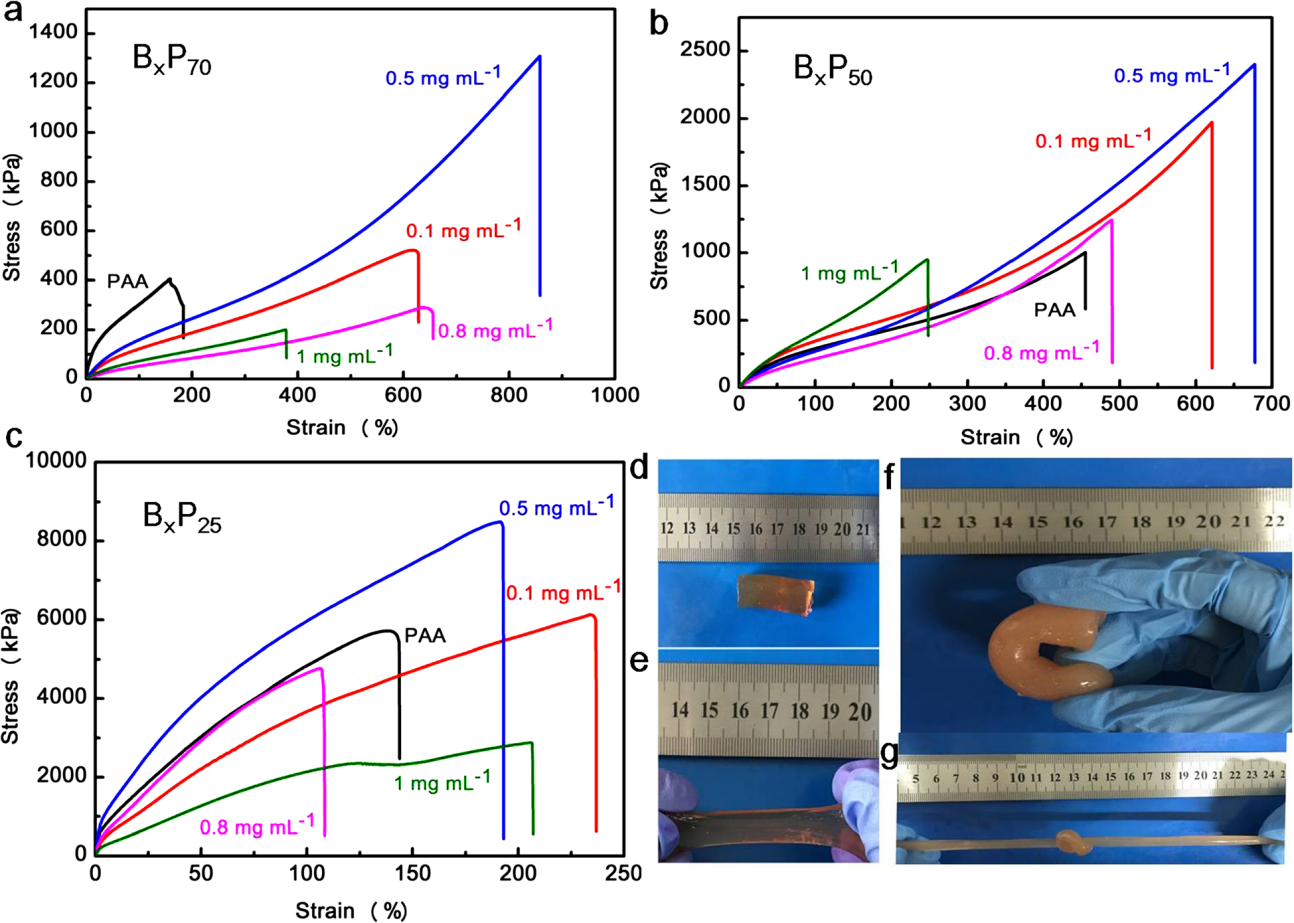
Tensile stress-strain curves of the composite hydrogels: **a** B_x_P_70_, **b** B_x_P_50_, and **c** B_x_P_25_. The digital photograph of composite hydrogels: **d** original, **e** stretched, **f** bent, and **g** stretched after being knotted

After drying, the morphologies of these hydrogels were observed by SEM. Porous structures have been observed in PAA hydrogel (Fig. 2a, b) and B_x_P_y_ composite hydrogel (Fig. 2c). The larger pores have an average diameter of around dozens of micrometers and the smaller pores have an average diameter in nanoscale. The presence of pores might provide high stretchability and squeezability to the hydrogels [35]. Compared to PAA hydrogels, the pores within the composite hydrogel are more isolated and uniform in sizes (Fig. 2c). And the BNNSs-NH_2_ can be observed in a SEM image (Fig. 2d) of hydrogel containing BNNSs-NH_2_ and were pointed out by arrows, and the insert image further verified the presence of the BNNS-NH_2_ [34]. To understand the effects of the two-type interactions, tensile tests of composite hydrogels with different BNNS-NH_2_ concentrations were conducted, and results from these hydrogels with different water contents are shown in Fig. 3a–c. Without BNNS-NH_2_, the fracture stress of B_0_P_70_ hydrogel was about 406 kPa, and the facture stress of B_0.1_P_70_ increased to 526 kPa by introducing a small amount of BNNS-NH_2_. The B_0.5_P_70_ exhibits a fracture stress of 1311 kPa, almost three times to B_0_P_70_ and two times to B_0.1_P_70_, as shown in Fig. 3a. The result far exceeded previous composite hydrogels in published work [34]. This means that the hydrogen bond formed between –COOH of PAA and –NH_2_ of BNNS-NH_2_ significantly enhanced the mechanical properties [36]. However, the fracture stress decreased when the BNNS-NH_2_ concentration continued to increase. The fracture stress becomes even lower than that of B_0_P_70_, which indicated that a prime balance of metal coordination interactions and hydrogen bonds was achieved at the BNNS-NH_2_ concentration of 0.5 mg mL^− 1^. Correspondingly, the greatest fracture stresses were also realized at the same BNNS-NH_2_ concentration (0.5 mg mL^− 1^) within composite hydrogels with the water content of 50 wt% and 25 wt% (Fig. 3b, c) which was an important factor to affect the mechanical properties of the hydrogel [37, 38]. The fracture stresses of the composite hydrogels were remarkably improved (Additional file 1: Figure S1-S5) when water content decreased to 50 wt% and to 25 wt%, attributed to the narrow space between chains at low water content [35]. Notably, the B_x_P_y_ hydrogels were able to withstand tensile, knotting, bending, and torsion even under high degree of deformations (Fig. 3d–g, Additional file 1: Figure S6). The addition of BNNS-NH_2_ might slightly change the pH of the solution due to the presence of –NH_2_ groups, leading to a change in association constant between –COOH and Fe^3+^. The balance between metal coordination interactions and hydrogen bonds was dependent on the BNNS-NH_2_ concentration with the constant content of Fe^3+^. The excellent mechanical behaviors of the composite hydrogel stemmed from the optimum balance between the metal coordination interaction in molecular scale and the hydrogen bond interactions in nanoscale [36].

**Fig. 4:**
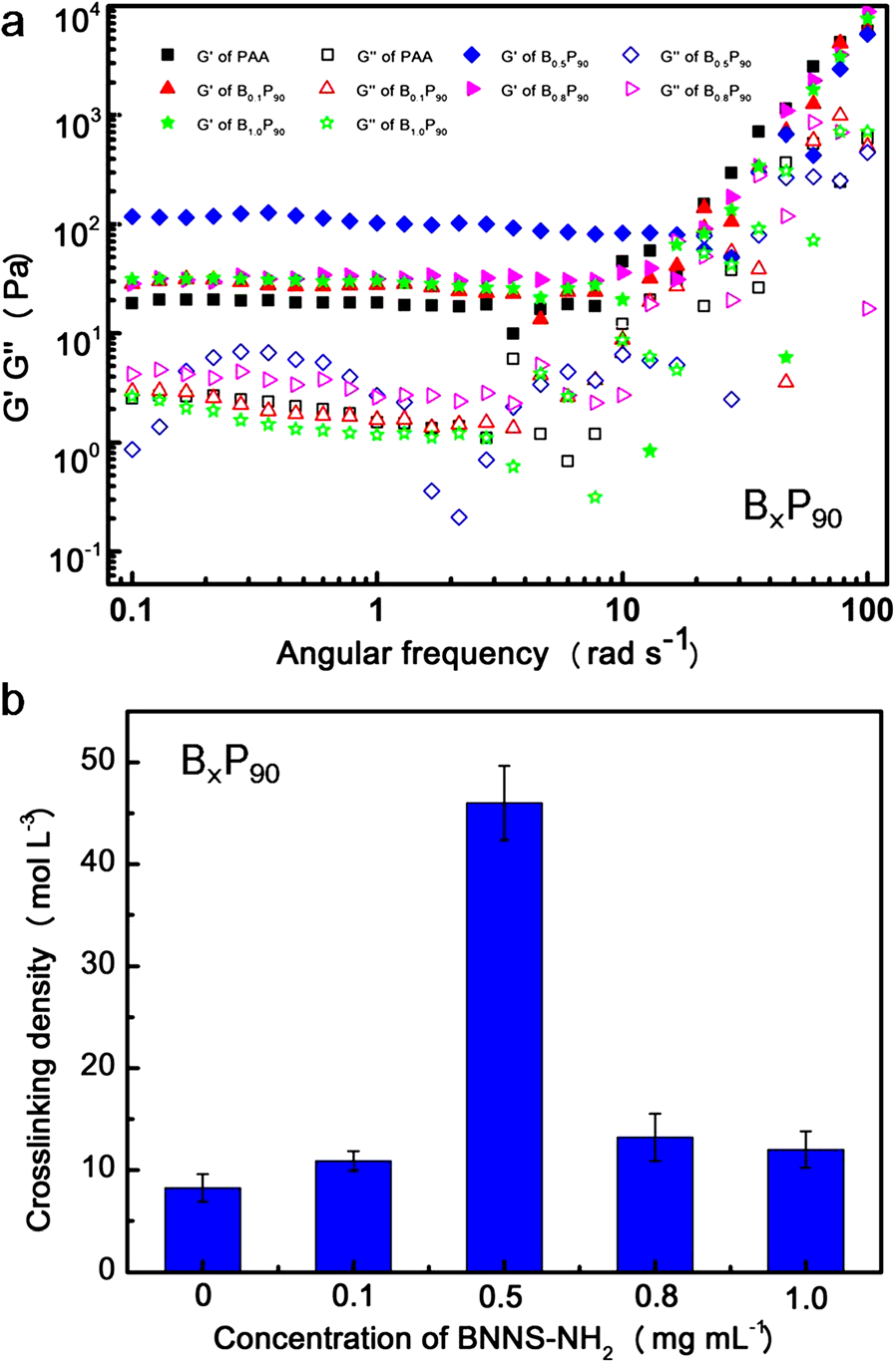
**a** Frequency dependence of storage moduli (G’) and loss moduli (G”) of B_x_P_90_ at a strain of 0.1%. **b** Crosslinking density of B_x_P_90_ calculated by equilibrium shear modulus

The effects of BNNS-NH_2_ concentration on mechanical properties of B_x_P_90_ were studied by rheological measurement (Fig. 4a). For all the samples, their storage moduli (G’) are always higher than the corresponding loss moduli (G”) in the frequency range from 0.1 to 100 rad s^− 1^, indicating the formation of three-dimensional networks [34, 36]. With the increase of frequency, both G’ and G” increased, but the increase in G” is more sharp, showing their shear-thin behaviors [34]. B_0.5_P_90_ exhibited the highest G’, which is consistent with the results from the tensile tests. From the equilibrium shear modulus (*G*_*e*_), crosslinking density (*N*) of these hydrogels can be calculated by using formula 1 [39–41].

1 $$ N= Ge/(RT) $$

Here, *G*_*e*_, *R*, and *T* are the terrace value of G’, gas constant, and absolute temperature, respectively. The crosslinking density is shown in Fig. 4b. With increase in the concentration of BNNS-NH_2_, the crosslinking density increased, which demonstrates that BNNS-NH_2_ also served as a crosslinker in the composite hydrogels through hydrogen bond interactions between –COOH of PAA and –NH_2_ of BNNS-NH_2_. However, the crosslinking density decreased when the BNNS-NH_2_ concentration is over 0.5 mg mL^− 1^ which corresponded with the results of the mechanical properties [40]. It is illustrated that the excess BNNS-NH_2_ leads to reunion of the nanosheets which impairs the enhancement to the composite hydrogels such as B_0.8_P_y_ and B_1.0_P_y_ [41, 42].

**Fig. 5:**
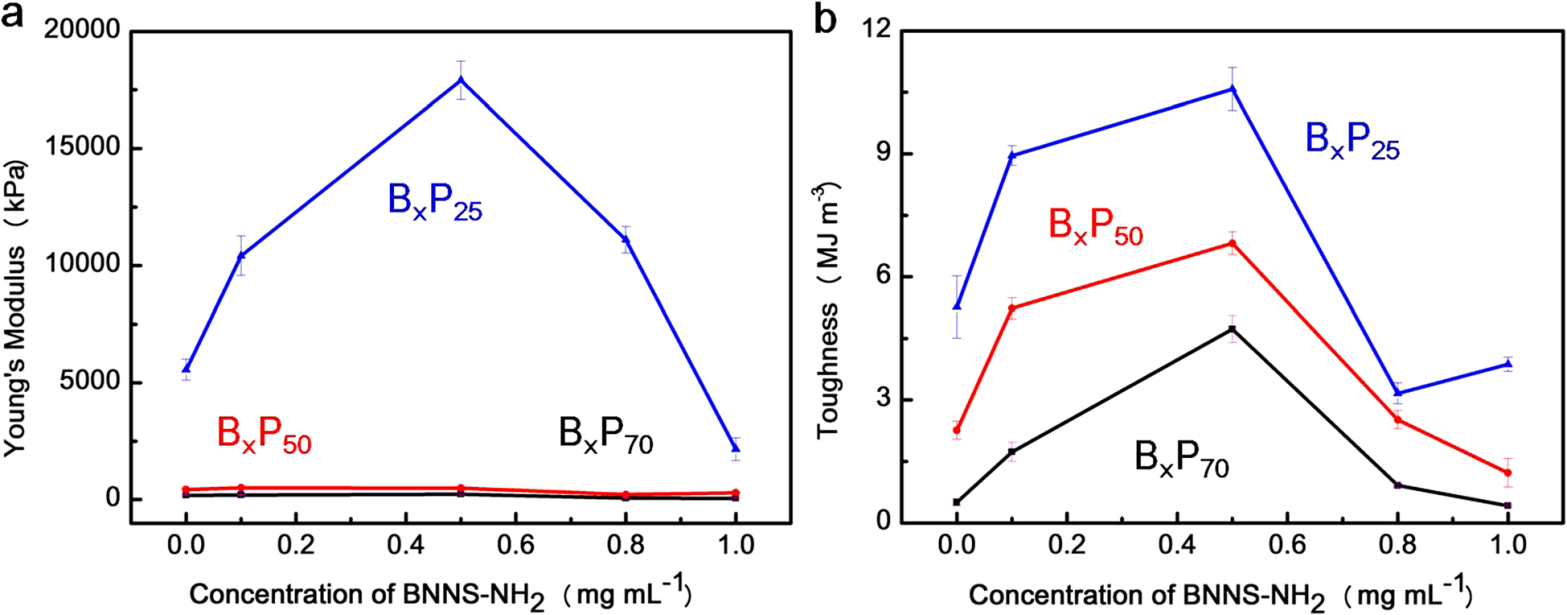
**a** Young’s modulus and **b** toughness of composite hydrogels with different water contents and BNNS-NH_2_ concentrations

To obtain hydrogels with excellent mechanical properties, the optimal balance of hydrogen bond interactions and metal coordination interactions can be achieved by adjusting the BNNS-NH_2_ concentrations while the content of Fe^3+^ is constant. Toughness and Young’s modulus representing stiffness are shown in Fig. 5a and Fig. 5b, respectively [24, 36, 37]. From Fig. 5a, hydrogels became stiff with decreasing water content or increasing BNNS-NH_2_ concentration till 0.5 mg mL^− 1^ consistent with the results of tensile test (Additional file 1: Figure S7).

The toughness is observed in Fig. 5b. It is clear that toughness increases with decreasing water content, similar to the trend of Young’s modulus. Without BNNS-NH_2_, the toughness of B_0_P_70_ was about ~ 0.5 MJ m^− 3^, and with BNNS-NH_2_, the toughness of B_0.5_P_70_ increased to ~ 4.7 MJ m^− 3^, almost eight times to that of B_0_P_70_. The B_0.5_P_25_ exhibited the highest Young’s modulus of ~ 17.9 MPa, highest tensile strength of ~ 8491 kPa, and highest toughness of ~ 10.5 MJ m^− 3^, which is far higher than that of B_0_P_25_.

The stiffness of most polymer hydrogels decreases with increase in the corresponding toughness. According to the Lake-Thomas model [42, 43], toughness increases but stiffness decreases with decreasing crosslinking density. In this work, a novel type hydrogel with both high stiffness and high toughness (B_0.5_P_y_) (Fig. 5) has been fabricated, which is different from the conventional hydrogels (high stiffness/low toughness or low stiffness/high toughness). The exceptional properties can be ascribed to the existence of hierarchical interactions: metal coordination interactions in molecular scale and hydrogen bonds in nanoscale.

**Fig. 6:**
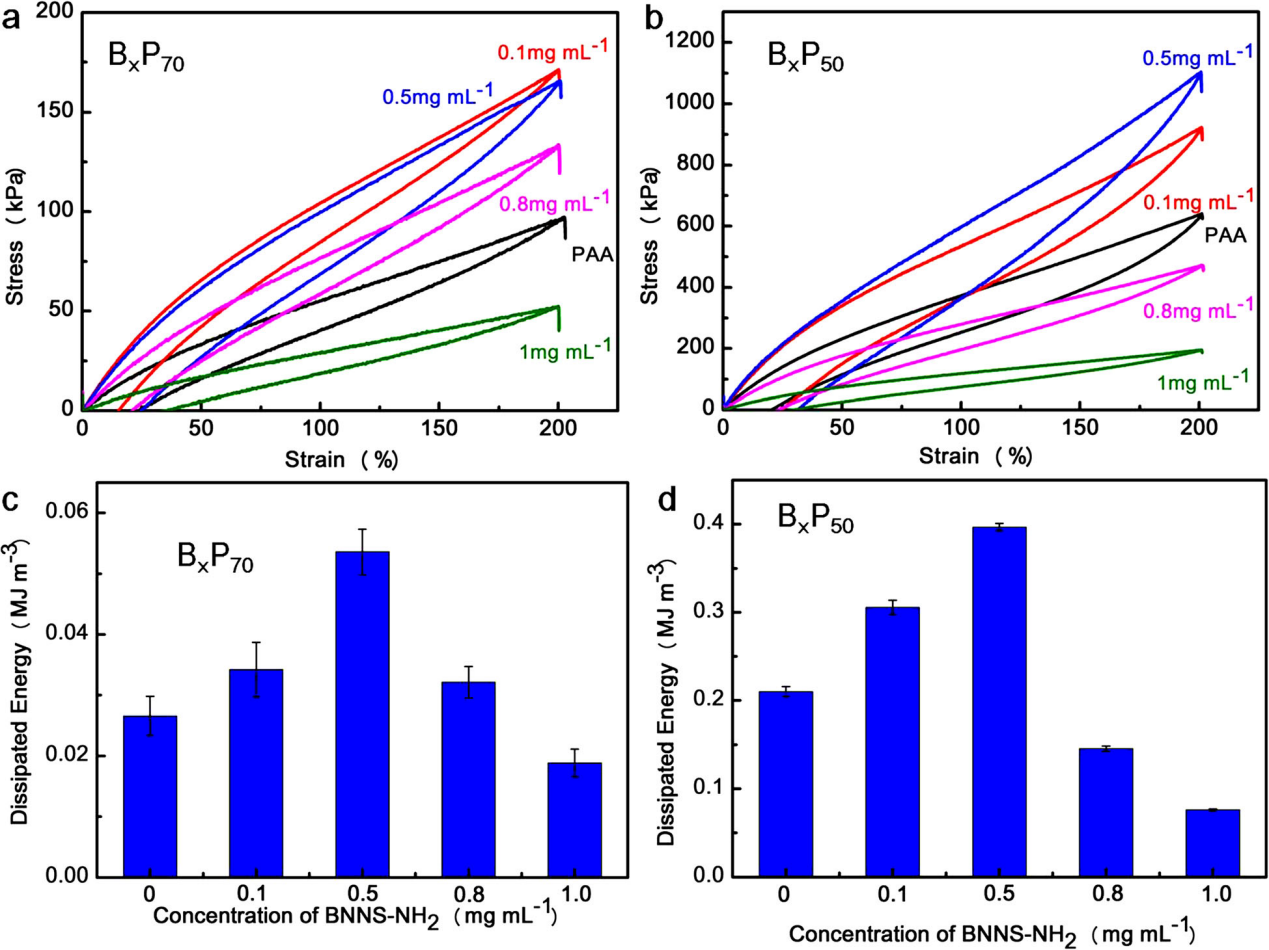
Cyclic tensile of loading-unloading curves of **a** B_x_P_70_ and **b** B_x_P_50_; the dissipated energy of **c** B_x_P_70_ and **d** B_x_P_50_

Cyclic tensile tests of B_x_P_70_ and B_x_P_50_ were conducted at the strain of 200% (Fig. 6a, b). Obvious hysteresis loops were observed for B_x_P_70_ and B_x_P_50_, and B_x_P_50_ showed much larger hysteresis loops, indicating the water content determining energy dissipation owing to more hydrogen bonds being established between polymer chains because of the shrunken networks [37]. The dissipated energy increased with increasing BNNS-NH_2_ concentration, and the maximum value was obtained at the concentration of 0.5 mg mL^− 1^ due to the establishment of numerous hydrogen bonds between BNNS-NH_2_ and PAA chains [34]. However, the dissipated energy decreased when BNNS-NH_2_ concentration increased to 0.8 and to 1.0 mg mL^− 1^, owing to the high concentration of BNNS-NH_2_ leading to aggregation of the nanosheets [41, 42]. This explanation is also suitable for specific stress-strain curves and rheology results of the B_x_P_y_ hydrogels.

**Fig. 7:**
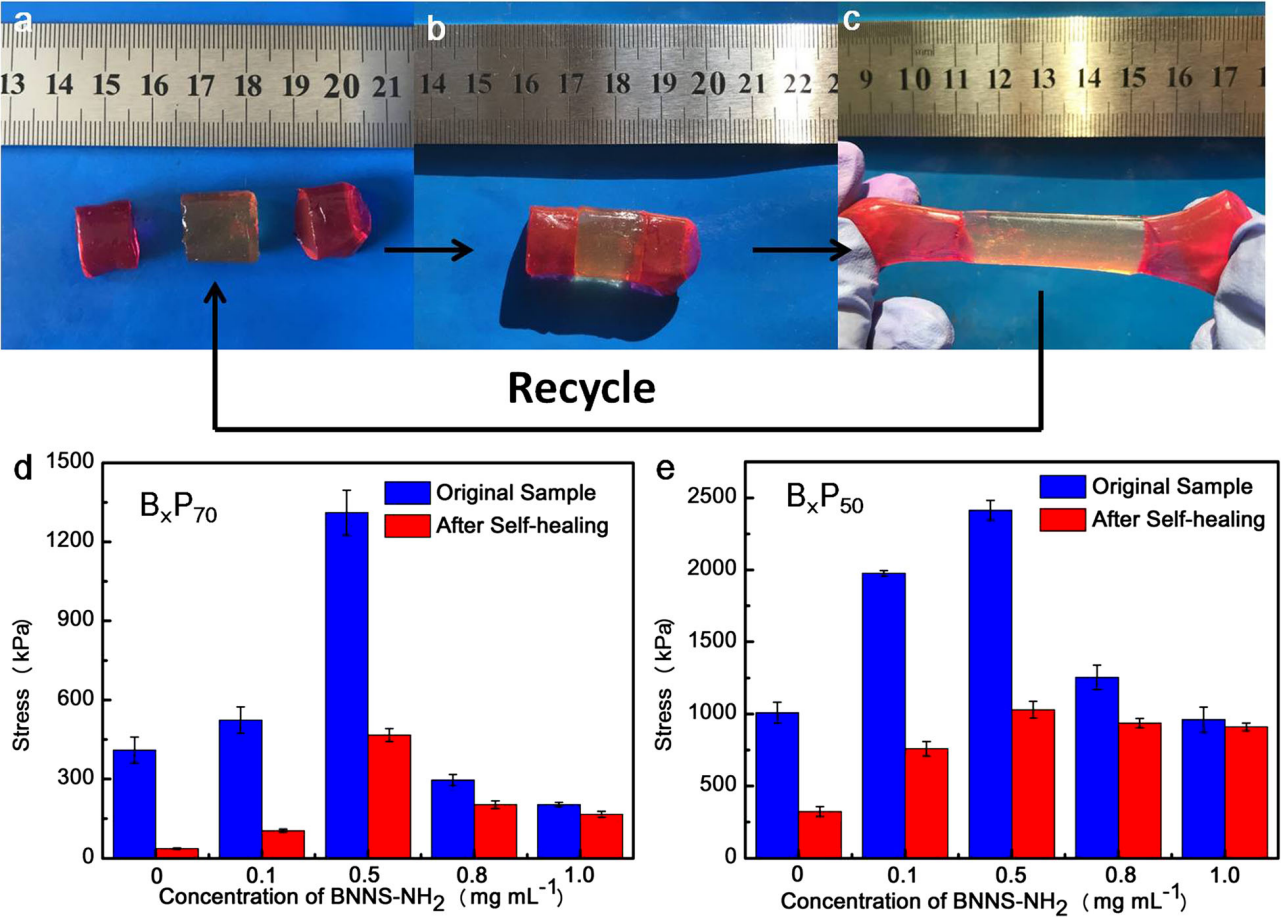
Self-healing process of B_0.5_P_90_ (**a**) the hydrogel was cut to three parts (the terminal two parts were dye to red by Rhodamine B). **b** Strictly put the three parts together one by one. **c** After 10 min healing, the healed hydrogel can be stretched. **d** The fracture stresses of original B_x_P_70_ and healed B_x_P_70_. **e** The fracture stresses of original B_x_P_50_ and healed B_x_P_50._ The healing time was 1 h

The rapid self-healing process can be realized without any external stimulus based on the abundant physical interactions: metal coordination interactions and hydrogen bonds. As shown in Fig. 7a–c, the original hydrogel was cut to three parts (two parts were dye to red by Rhodamine B to distinguish the scars of the damaged hydrogel) and then they were contacted at the damaged surfaces. Without any external stimulus, the cut hydrogel contacted for 10 min at room temperature, the healed hydrogel can be stretched to certain strain. Self-healing efficiency can be calculated from the ratio of fracture stress of the healed hydrogels and the original hydrogels. Original B_0_P_70_ exhibited fracture stress of ~ 410 kPa, and the corresponding healed hydrogel exhibited a fracture stress of only ~ 37 kPa, indicating that the self-healing efficiency was only 9%. In comparison, the fracture stress of original and healed B_1_P_70_ were about ~ 203 kPa and ~ 166 kPa, respectively, and the self-healing efficiency is about 81%, which is significantly higher than the hydrogels without the BNNS-NH_2_. Similarly, as shown in Fig. 7e, the self-healing efficiency of B_0_P_50_ hydrogel was 31.8%, while the B_1_P_50_ hydrogel was 94.6%. This result indicates that the presence of nanoscale hydrogen bonds between BNNS-NH_2_ and PAA polymer chains in nanoscale enhanced the self-healing ability ascribing that the content of reversible bonds is the key influencing factor of the self-healing efficiency, and it is well known that the self-healing efficiency is proportional to the content of the reversible bonds [16, 21, 36, 42]. However, while the water content was reduced to 25 wt% (Additional file 1: Figure S8), the self-healing efficiency declined sharply, because the movement of Fe^3+^ was impeded at such a low water content. This assumption was confirmed by the fact that healing efficiency of B_x_P_90_ was much better than other water content, with only 10 min required attributed to the reason that higher water contents make the Fe^3+^ to migrate easily and re-establish hydrogen bonds readily [36].

## Conclusions

In summary, the novel composite hydrogels have been fabricated through hierarchically physical interactions: the metal coordination interaction in molecular scale and hydrogen bond in nanoscale. The hydrogels exhibit enhanced stiffness (about 17.9 MPa), toughness (about 10.5 MJ m^− 3^), extension, and self-healing ability. The reversibility of metal coordination interaction and hydrogen bond interaction is responsible for the enhanced mechanical properties and self-healing efficiency. Combined with facile preparation, enhanced mechanical properties and self-healing ability make these composite hydrogels suitable for many potential applications.

## Additional file

Additional file 1: **Figure S1.** Tensile stress-strain curves of B_0_P_y_ hydrogels with different water contents. **Figure S2.** Tensile stress-strain curves of B_0.1_P_y_ hydrogels with different water contents. **Figure S3.** Tensile stress-strain curves of B_0.5_P_y_ hydrogels with different water contents. **Figure S4.** Tensile stress-strain curves of B_0.8_P_y_ hydrogels with different water contents. **Figure S5.** Tensile stress-strain curves of B_1.0_P_y_ hydrogels with different water contents. **Figure S6.** The digital photograph of torsion hydrogel. **Figure S7.** Young’s modulus of hydrogels of B_x_P_50_ and B_x_P_70_. **Figure S8.** the fracture stresses of original B_x_P_25_ hydrogels and healed B_x_P_25_ hydrogels with different BNNS-NH_2_ concentrations (the healing time was 1 h). (DOCX 783 kb)

## Abbreviations

B_x_P_y_: Composite hydrogel with the BNNS-NH_2_ concentration of *x* mg mL^− 1^ and water content of *y* wt%
Fe^3+^: Ferric ion
FTIR: Fourier-transform infrared
PAA/BNNS-NH_2_: Poly(acrylic acid)/surface-modified boron nitride nanosheets
PAA/GO: Graphene oxide/poly(acrylic acid)
SEM: Scanning electronic micrographs
