# Supplementary material for: Fabrication of Poly(acrylic acid)/Boron Nitride Composite Hydrogels with Excellent Mechanical Properties and Rapid Self-Healing Through Hierarchically Physical Interactions
Source: Nanoscale Res Lett. 2018 Dec 5;13:393. doi: 10.1186/s11671-018-2800-2 (PMC6281544; doi:10.1186/s11671-018-2800-2)
Supplement: Supplementary file 1 — Figure S1. Tensile stress-strain curves of B0Py hydrogels with different water contents. Figure S2. Tensile stress-strain curves of B0.1Py hydrogels with different water contents. Figure S3. Tensile stress-strain curves of B0.5Py hydrogels with different water contents. Figure S4. Tensile stress-strain curves of B0.8Py hydrogels with different water contents. Figure S5. Tensile stress-strain curves of B1.0Py hydrogels with different water contents. Figure S6. The digital photograph of torsion hydrogel. Figure S7. Young’s modulus of hydrogels of BxP50 and BxP70. Figure S8. the fracture stresses of original BxP25 hydrogels and healed BxP25 hydrogels with different BNNS-NH2 concentrations (the healing time was 1 h). (DOCX 783 kb) [file 11671_2018_2800_MOESM1_ESM.docx]

Fabrication of poly([acrylic](javascript:void(0);) [acid](javascript:void(0);))/boron nitride composite hydrogels with excellent mechanical properties and rapid self-healing through hierarchically physical interactions

Shishan Xue^1^, Yuanpeng Wu ^1, 2*^, Meiling Guo^1^, Guanfei Liu^1^, Dan Liu^3^, Tao Zhang^4*^, Weiwei Lei^3*^


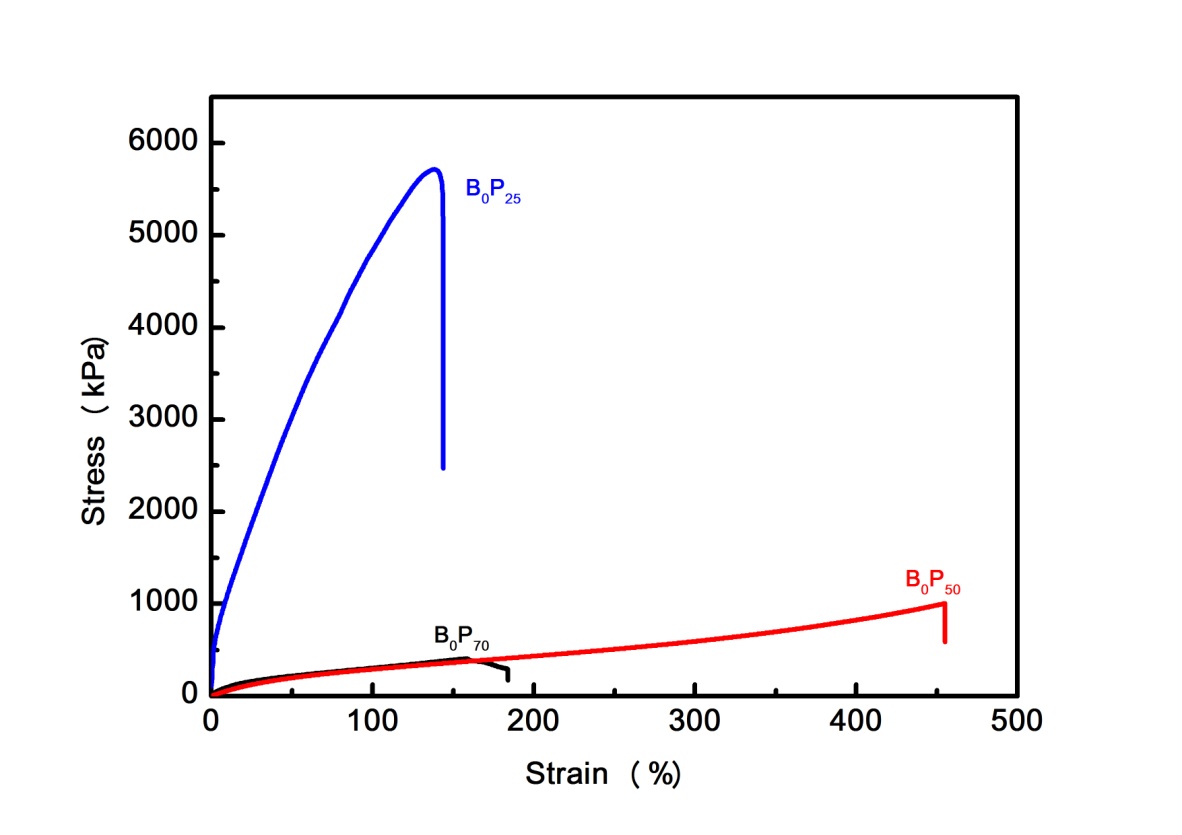


Figure S1 Tensile stress-strain curves of B_0_P_y_ hydrogel with different water content.


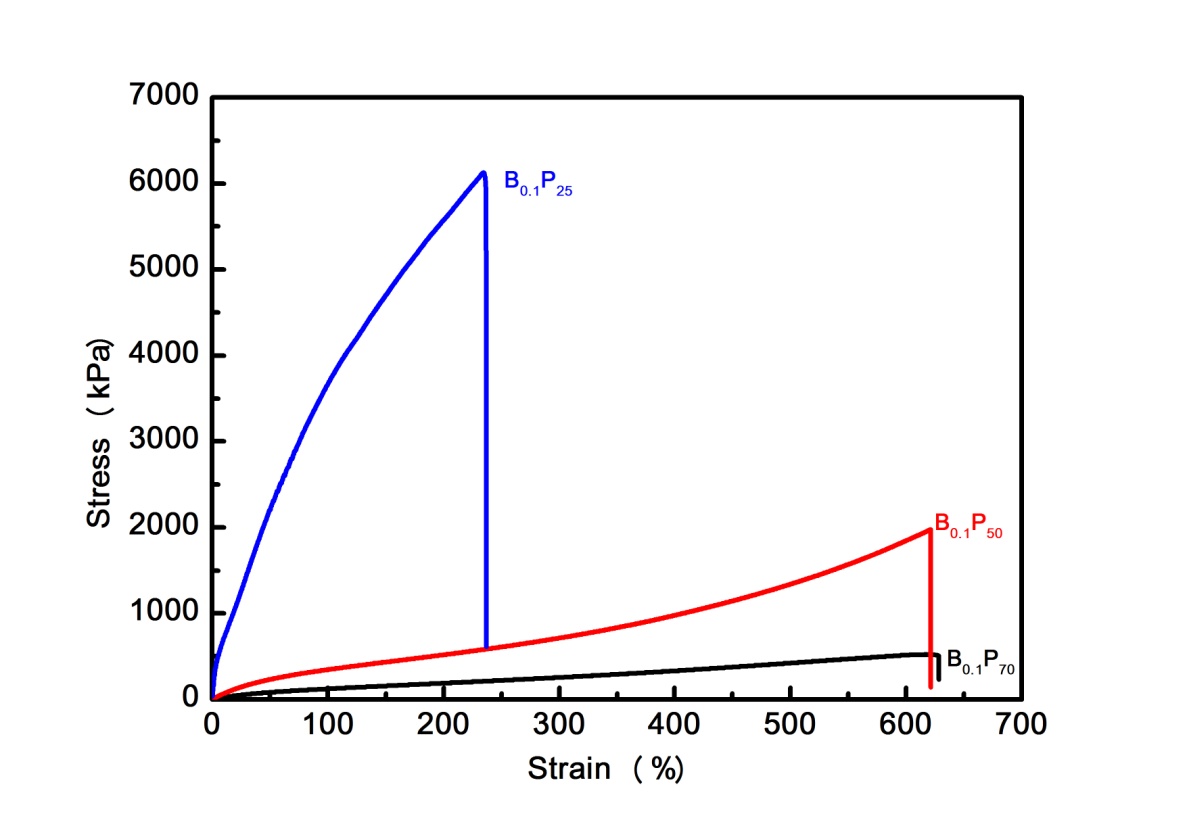


Figure S2 Tensile stress-strain curves of B0.1Py hydrogel with different water content.


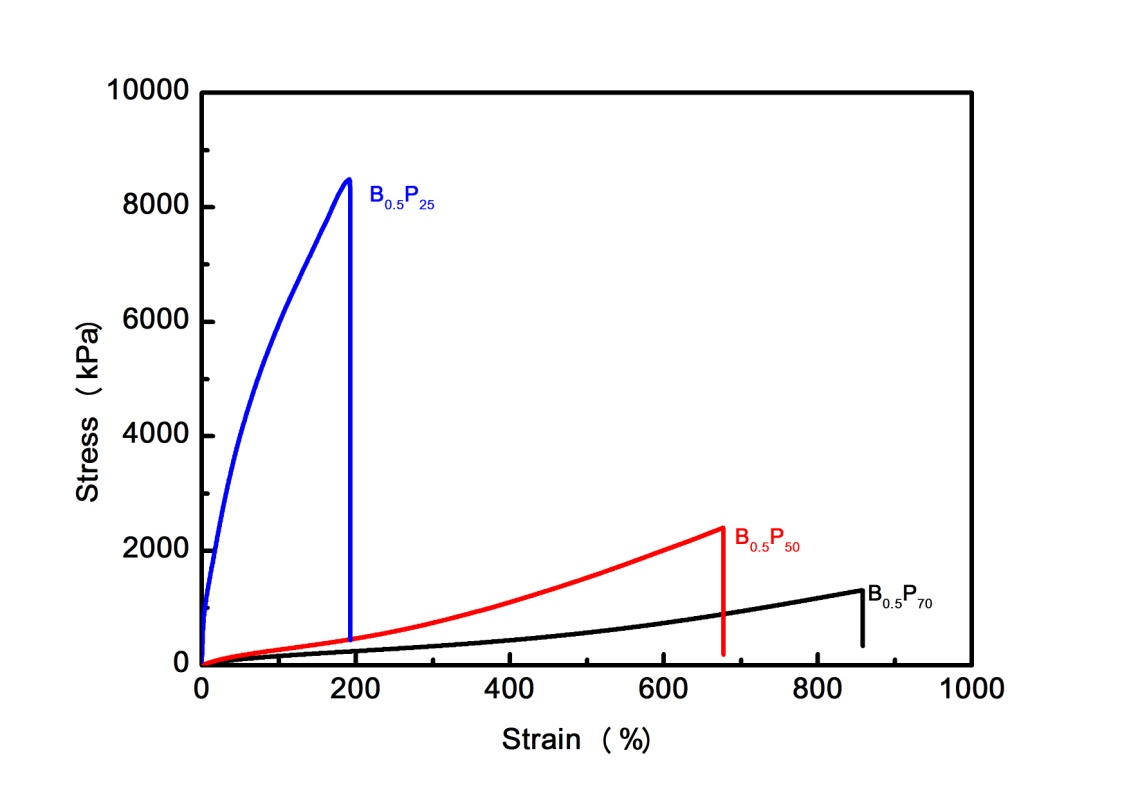


Figure S3 Tensile stress-strain curves of B_0.5_P_y_ hydrogel with different water content.


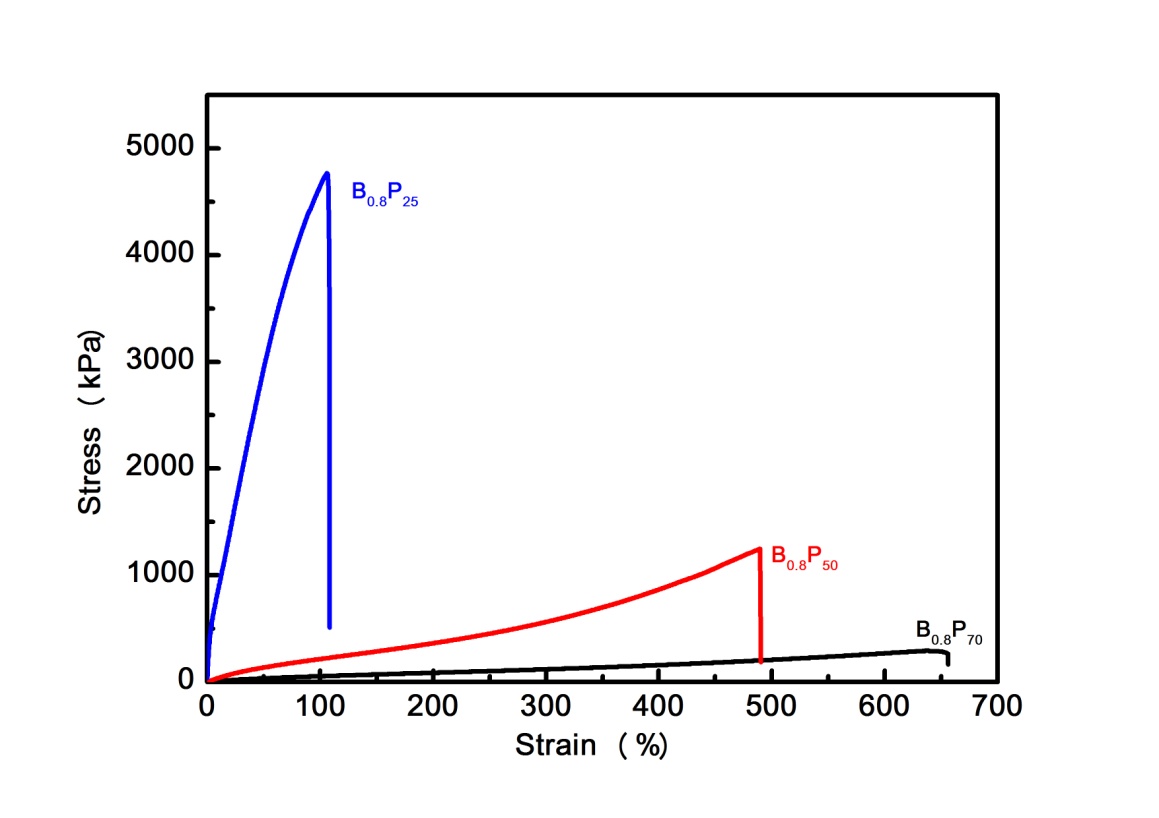


Figure S4 Tensile stress-strain curves of B_0.8_P_y_ hydrogel with different water content.


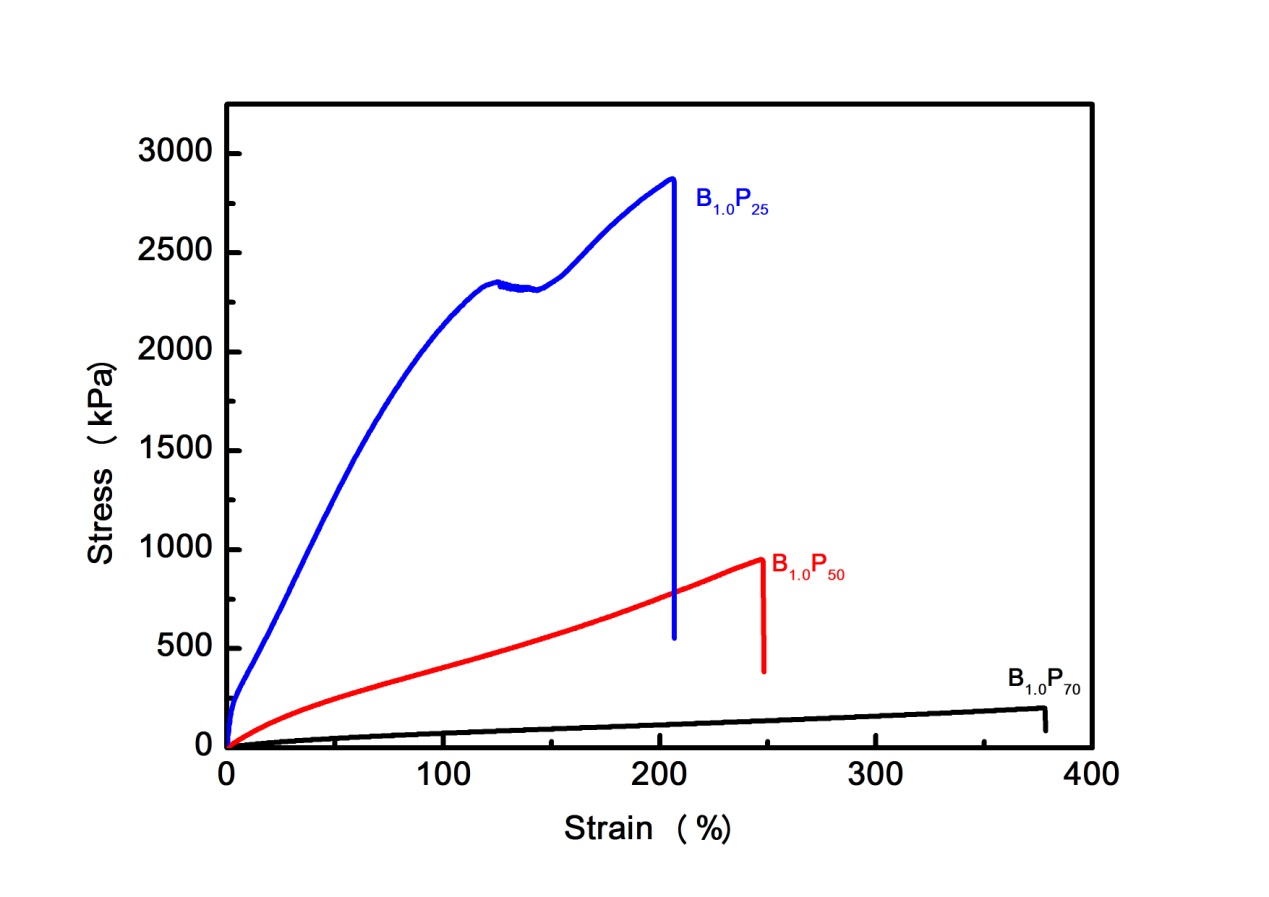


Figure S5 Tensile stress-strain curves of B_1.0_P_y_ hydrogel with different water content.


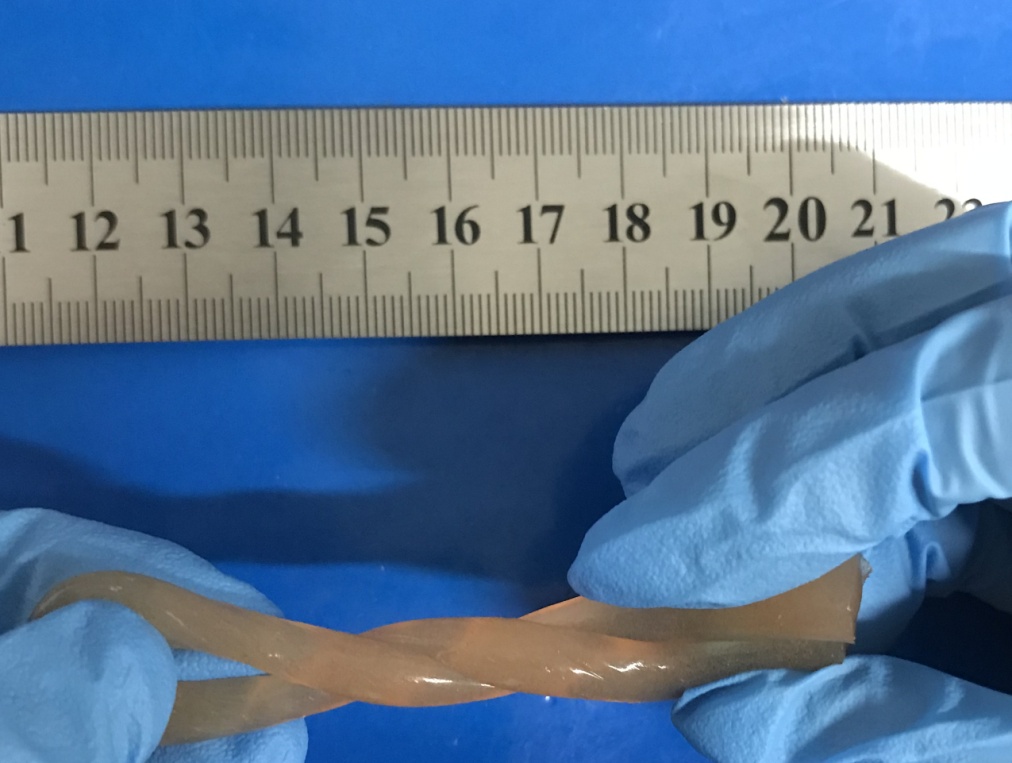


Figure S6 The digital photograph of torsion hydrogel.


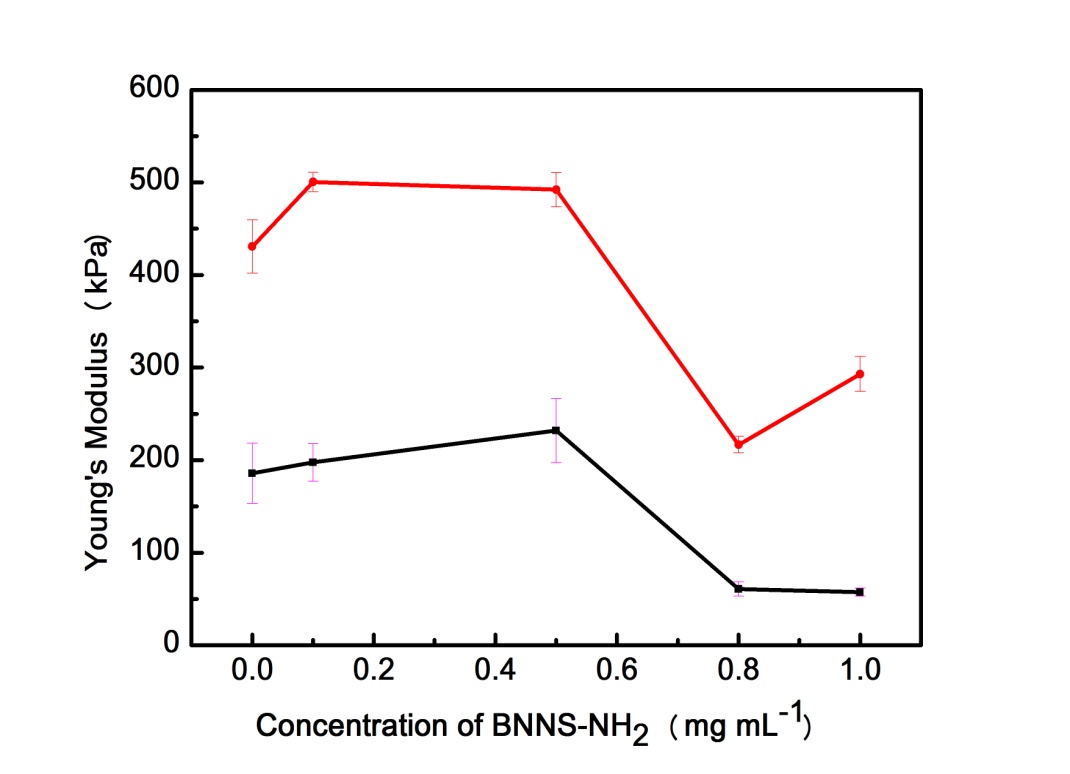


Figure S7 Young’s modulus of hydrogels of B_x_P_50_ and B_x_P_70_


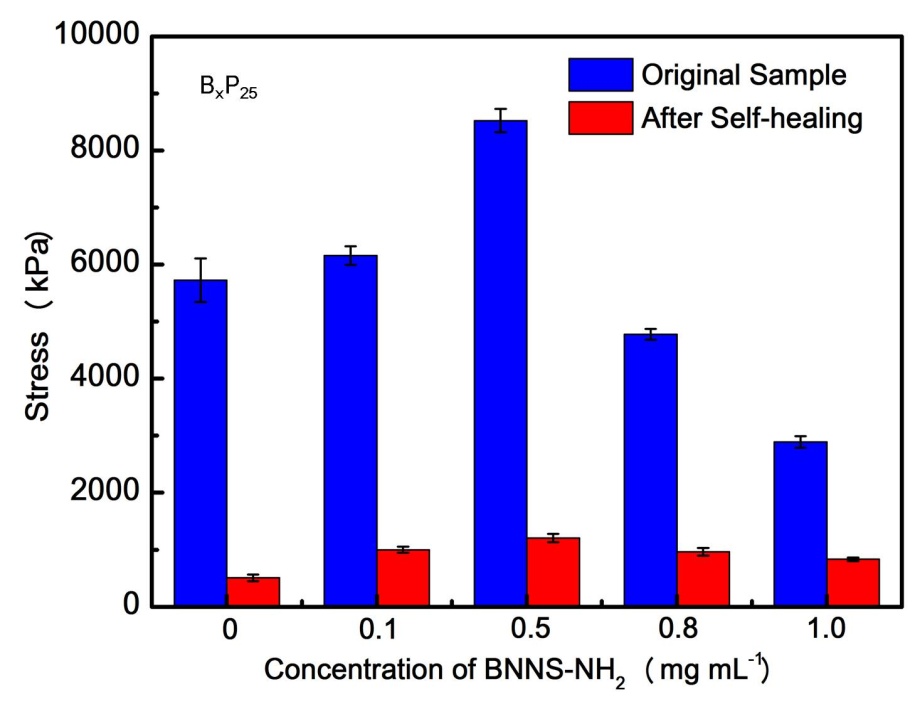


Figure S8 the fracture stress of original B_x_P_25_ hydrogels and healed B_x_P_25_ hydrogels with different BNNS concentration (the healing time was 1 h).
